# The Effect of Disease Modifying Therapies on Disease Progression in Patients with Relapsing-Remitting Multiple Sclerosis: A Systematic Review and Meta-Analysis

**DOI:** 10.1371/journal.pone.0144538

**Published:** 2015-12-07

**Authors:** Georgios Tsivgoulis, Aristeidis H. Katsanos, Nikolaos Grigoriadis, Georgios M. Hadjigeorgiou, Ioannis Heliopoulos, Panagiotis Papathanasopoulos, Constantinos Kilidireas, Konstantinos Voumvourakis, Efthimios Dardiotis, HELANI (Hellenic Academy of Neuroimmunology)

**Affiliations:** 1 Second Department of Neurology, “Attikon” Hospital, School of Medicine, University of Athens, Athens, Greece; 2 Department of Neurology, The University of Tennessee Health Science Center, Memphis, Tennessee, United States of America; 3 International Clinical Research Center, Department of Neurology, St. Anne's University Hospital in Brno, Brno, Czech Republic; 4 Department of Neurology, School of Medicine, University of Ioannina, Ioannina, Greece; 5 Second Department of Neurology, “AHEPA” University Hospital, Aristotelion University of Thessaloniki, Thessaloniki, Macedonia, Greece; 6 Department of Neurology, University Hospital of Larissa, University of Thessaly, Larissa, Greece; 7 Department of Neurology, Alexandroupolis University Hospital, Democritus University of Thrace, Alexandroupolis, Greece; 8 Department of Neurology, University of Patras Medical School, Patras, Greece; 9 First Department of Neurology, “Eginition” Hospital, School of Medicine, University of Athens, Athens, Greece; Friedrich-Alexander University Erlangen, GERMANY

## Abstract

**Importance:**

A number of officially approved disease-modifying drugs (DMD) are currently available for the early intervention in patients with relapsing-remitting multiple sclerosis (RRMS). The aim of the present study was to systematically evaluate the effect of DMDs on disability progression in RRMS

**Methods:**

We performed a systematic review on MEDLINE and SCOPUS databases to include all available placebo-controlled randomized clinical trials (RCTs) of RRMS patients that reported absolute numbers or percentages of disability progression during each study period. Observational studies, case series, case reports, RCTs without placebo subgroups and studies reporting the use of RRMS therapies that are not still officially approved were excluded. Risk ratios (RRs) were calculated in each study protocol to express the comparison of disability progression in RRMS patients treated with a DMD and those RRMS patients receiving placebo. The mixed-effects model was used to calculate both the pooled point estimate in each subgroup and the overall estimates.

**Results:**

DMDs for RRMS were found to have a significantly lower risk of disability progression compared to placebo (RR = 0.72, 95%CI: 0.66–0.79; p<0.001), with no evidence of heterogeneity or publication bias. In subsequent subgroup analyses, neither dichotomization of DMDs as “first” and “second” line RRMS therapies [(RR = 0.72, 95% CI = 0.65–0.80) vs. (RR = 0.72, 95% = 0.57–0.91); p = 0.96] nor the route of administration (injectable or oral) [RR = 0.75 (95% CI = 0.64–0.87) vs. RR = 0.74 (95% CI = 0.66–0.83); p = 0.92] had a differential effect on the risk of disability progression. Either considerable (5–20%) or significant (>20%) rates of loss to follow-up were reported in many study protocols, while financial and/or other support from pharmaceutical industries with a clear conflict of interest on the study outcomes was documented in all included studies.

**Conclusions:**

Available DMD are effective in reducing disability progression in patients with RRMS, independently of the route of administration and their classification as “first” or “second” line therapies. Attrition bias needs to be taken into account in the interpretation of these findings.

## Introduction

Multiple sclerosis (MS) is a chronic inflammatory and neurodegenerative disease that manifests with acute relapses and progressive disability [1]. Expanded Disability Status Scale (EDSS) change is the main outcome measure used in MS clinical studies [2], as a potential indicator of neurological improvement that correlates directly with the quality of patients' life [3]. In clinical practice EDSS progression is considered one of main indicators for change in treatment for MS patients with clinical deterioration [4], as it has been observed that increases in EDSS scale are independently associated with MS therapy cessation [5].

A number of officially approved disease-modifying drugs (DMD), including novel oral agents, are currently available for the aggressive early intervention in patients with relapsing-remitting MS (RRMS), promising higher treatment goals and long-term outcomes improvement [6]. Even though DMDs are considered to be equally effective in delaying EDSS progression in RRMS patients [7], observational study data report that both DMD choice and cumulative treatment duration may have a significant impact on EDSS change in patients with RRMS [8, 9].

The aim of the present meta-analysis was to systematically investigate the effect of all available DMDs on disability progression reduction in RRMS using follow-up data from all available placebo-controlled randomized clinical trials (RCT). Moreover, we sought to evaluate potential sources of heterogeneity regarding the potential differential effect of DMD subgroups on disability progression.

## Methods

### Trial identification and data abstraction

This meta-analysis is presented according to the Preferred Reporting Items for Systematic Reviews and Meta-Analyses (PRISMA) guidelines for systematic reviews and meta-analyses (S1 PRISMA Checklist) [10]. Eligible placebo-control RCTs that reported absolute numbers or percentages of RRMS patients with disability progression during the study period were identified by searching MEDLINE, SCOPUS and the CENTRAL Register of Controlled Trials. The following keywords were used in all database searches: “relapsing-remitting multiple sclerosis”, “RRMS”, “disability” and “EDSS change”. We imposed no language or other restrictions. Last literature search was performed on February 7th, 2015. We examined reference lists of all retrieved articles to identify studies that may have been missed by the initial database search.

Database search was performed independently by three reviewers (GT, ED & AHK) to include only placebo-control RCTs that reported either the absolute or the percent numbers of RRMS patients with disability progression during the study period in both treatment and placebo subgroups. We excluded from the quantitative/qualitative analysis all: 1.Observational studies, 2.case series, 3.case reports, 4.RCTs without placebo subgroups and 5.studies reporting the use of RRMS therapies that are not still officially approved. Emerging disagreements regarding the literature search results between the three coauthors, were resolved with consensus [6].

In each eligible study we used a predefined 7-point quality control to address for biases. For each quality item the corresponding risk of bias was categorized as low, high or unclear according to the suggestions by Higgins et al [11, 12]. Complete outcome data were judged as "low risk" when the percentage of participants lost to follow-up was lower than 5% and "high risk" when the reported loss to follow up was more than 20%. In studies reporting loss to follow up between 5%-20% the risk of attrition bias was categorized as "unclear" [13]. In the “other bias” category we included all other potential sources of bias, including the source of funding reported in each protocol [11, 14]. Bias identification within studies was independently performed by the three reviewers who performed the literature search. (GT, AHK, ED). All emerging conflicts in quality control were resolved with consensus.

Absolute or percent numbers of RRMS patients with disability progression during the study period were extracted independently after bias identification by the same authors (GT, ED & AHK). The active treatment arm with the finally approved dose of DMD was selected in each trial for comparisons versus the placebo arm.

### Statistical analyses

We calculated Risk ratios (RRs) in each study protocol to express the comparison of disability progression in RRMS patients treated with a DMD and those RRMS patients receiving placebo. RR values smaller than 1 denote that the treatment under investigation has a positive effect in the number of RRMS patients with disability progression compared to placebo. A random-effects model (DerSimonian Laird) was used to calculate the pooled RRs. The equivalent z test was performed for each pooled RR, and if p < 0.05 it was considered statistically significant [11].

We assessed heterogeneity between studies with the Cochran Q and I^2^ statistics. For the qualitative interpretation of heterogeneity, I^2^ values of at least 50% were considered to represent substantial heterogeneity, while values of at least 75% indicated considerable heterogeneity, as per the Cochrane Handbook [11, 15]. We evaluated publication bias both graphically using a funnel plot [16] and with the Egger’s statistical test for funnel plot asymmetry [17].

After the main analysis, we conducted predefined subgroup analyses according to (i) current categorization of eligible DMDs as “first line” (INFb-1b, peginterferon beta- 1a, glatiramer acetate, INFb-1a, teriflunomide, dimethyl fumarate) and “second line” (natalizumab & fingolimod) RRMS treatments (ii) the DMT route of administration: injectable subcutaneously (IFNβ-1a, IFNβ-1b, peginterferon beta- 1a and glatiramer acetate) or intramusculary (IFNβ-1a) vs. oral (fingolimod, teriflunomide, dimethyl fumarate).

We used the mixed-effects model was used to calculate both the pooled point estimate in each subgroup and the overall estimates [11]. According to the mixed-effects model, a random effects model was first used to combine studies within each subgroup and then a fixed effect model was used to combine subgroups and estimate the overall effect. We assumed the study-to-study variance (tau-squared) to be the same for all subgroups. Tau-squared was first computed within subgroups and then pooled across subgroups [11].

Finally, we performed univariate post-hoc meta-regression analyses, using the random effects model (Method of Moments), to evaluate reported study duration as a possible moderator of the percentage of patients with disability progression.

Statistical analyses were conducted using Review Manager (RevMan) Version 5.3 software (Copenhagen: The Nordic Cochrane Centre, The Cochrane Collaboration, 2014) and Comprehensive Meta-analysis Version 2 software (Borenstein M, Hedges L, Higgins J, Rothstein H, Biostat, Englewood NJ, 2005).

## Results

### Study selection and study characteristics

**Fig 1:**
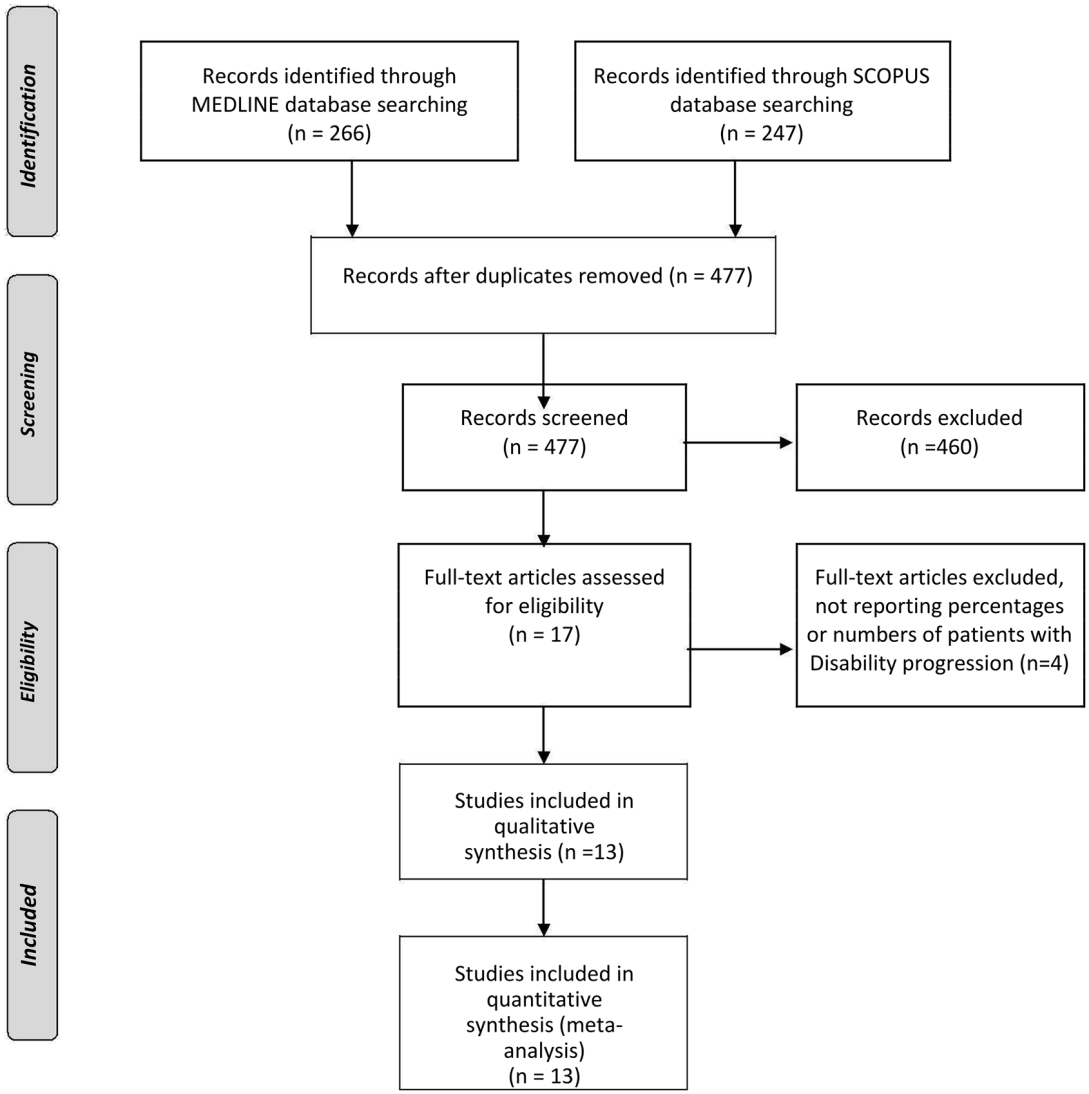
Flow chart presenting the selection of eligible studies.

Database search of MEDLINE and SCOPUS yielded 266 and 247 results respectively. No additional RCTs were identified in the CENTRAL Register of Controlled Trials database. After removing duplicate studies, the titles and abstracts from the remaining 477 studies were screened and **17** potentially eligible studies for the meta-analysis were retained. After retrieving the full-text version of the aforementioned **17** studies, 4 studies were excluded because they provided neither percentages nor numbers of patients with disability progression during the study period [18–21]. No disagreement about the literature search results emerged between the three reviewers and the 13 studies that met the predefined inclusion/exclusion criteria were included both in the qualitative and quantitative synthesis (Fig 1) [22–34]. The characteristics of the included studies (9788 total RRMS patients) are summarized in S1 Table. The following treatment arms (including only placebo arms and active arms with approved doses of available DMD) of the 13 selected RCT were included in the present analyses: INFb-MS (INFβ-1b 0.25mg/ml subcutaneous) [22], Copolymer (glatiramer acetate 20mg/ml subcutaneous) [23], MSCRG (INFβ-1a 30mcg/ml intramuscular) [24], PRISMS (INFβ-1a 22mcg/0.5ml or 44mcg/0.5ml subcutaneous) [25], AFFIRM (natalizumab 20mg/ml intravenous) [26], FREEDOMS I (fingolimod 0.5mg/cap per os) [27], FREEDOMS II (fingolimod 0.5mg/cap per os) [28], TEMSO (teriflunomide 14mg/tab per os) [29], TOWER (teriflunomide 14mg/tab per os) [30], CONFIRM (dimethyl fumarate 240mg/cap) [31], DEFINE (dimethyl fumarate 240mg/cap) [32], GALA (glatiramer acetate 40mg/ml subcutaneous) [33], ADVANCE (peginterferon beta-1a 125 μg/ml subcutaneously) [34]. The duration of studies varied from 1 year to 3 years. One year follow-up was reported in **5** study protocols [23, 27, 28, 33, 34], approximately 1,5 year follow-up in one study protocol [30], two year follow-up in 4 studies [24, 20, 31, 32], approximately 2,5 years in one study [26] and three year follow-up in two studies [22, 29]. In all studies, except for one [24], disability progression was assessed at 3-months.

### Risk of bias for independent studies

**Fig 2:**
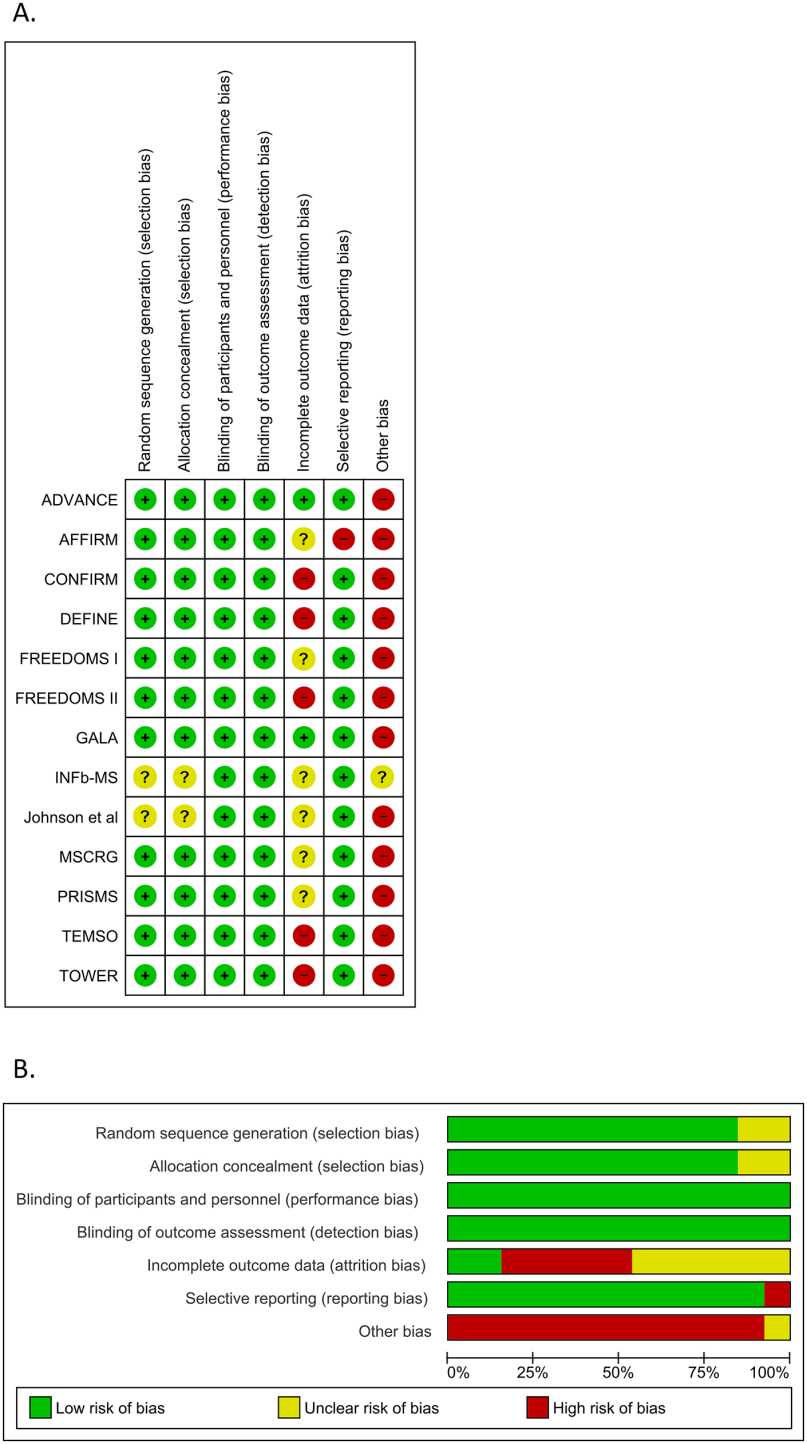
**A) Risk of bias summary**. Review authors' judgments about each risk of bias item for each included study. **B) Risk of bias graph**. Review authors' judgments about each risk of bias item presented as percentages across all included studies.

Risk of bias in the included studies is summarized in Fig 2A and 2B. Random sequence generation and allocation concealment was adequately reported in all trials, except for two [22, 23]. Blinding of participants, personnel and outcome assessment was sufficient in all protocols. Six of the study protocols reported loss to follow up percentages between 5%-20% [22–27], 5 studies reported loss to follow up more than 20% of the baseline number of participants [28–32] and the remaining 2 studies reported losses to follow-up less than 5% [33, 34]. Selective reporting bias was detected in only one study [26]. All study protocols were supported financially partly [23, 24] or solely [25–32, 33, 34] by the pharmaceutical companies that produce and market the drug under consideration in each study. Funding sources were not reported in the disclosures of one study protocol [22], providing thus insufficient information to permit judgment.

### Overall analysis and subgroup analyses

**Fig 3:**
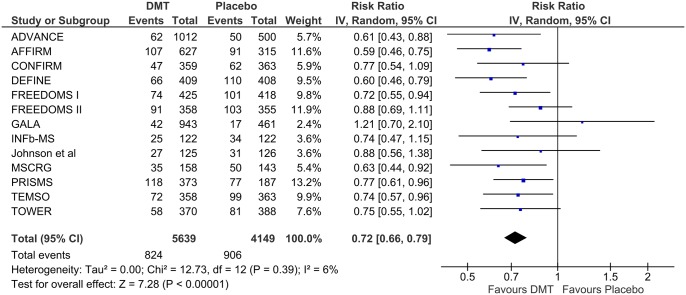
Overall analysis of disability progression in placebo-control randomized clinical trials of different disease modifying therapies in patients with relapsing-remitting multiple sclerosis.

Patients receiving approved DMDs for RRMS were found to have a significantly lower risk of disability progression compared to those receiving placebo (RR = 0.72, 95%CI: 0.66–0.79; p<0.001; Fig 3). No evidence of heterogeneity was found between estimates (I^2^ = 6%, p = 0.39). Moreover, no evidence of publication bias was detected in the funnel plot inspection (S1 Fig) or in the Egger’s statistical test (p = 0.178).

**Fig 4:**
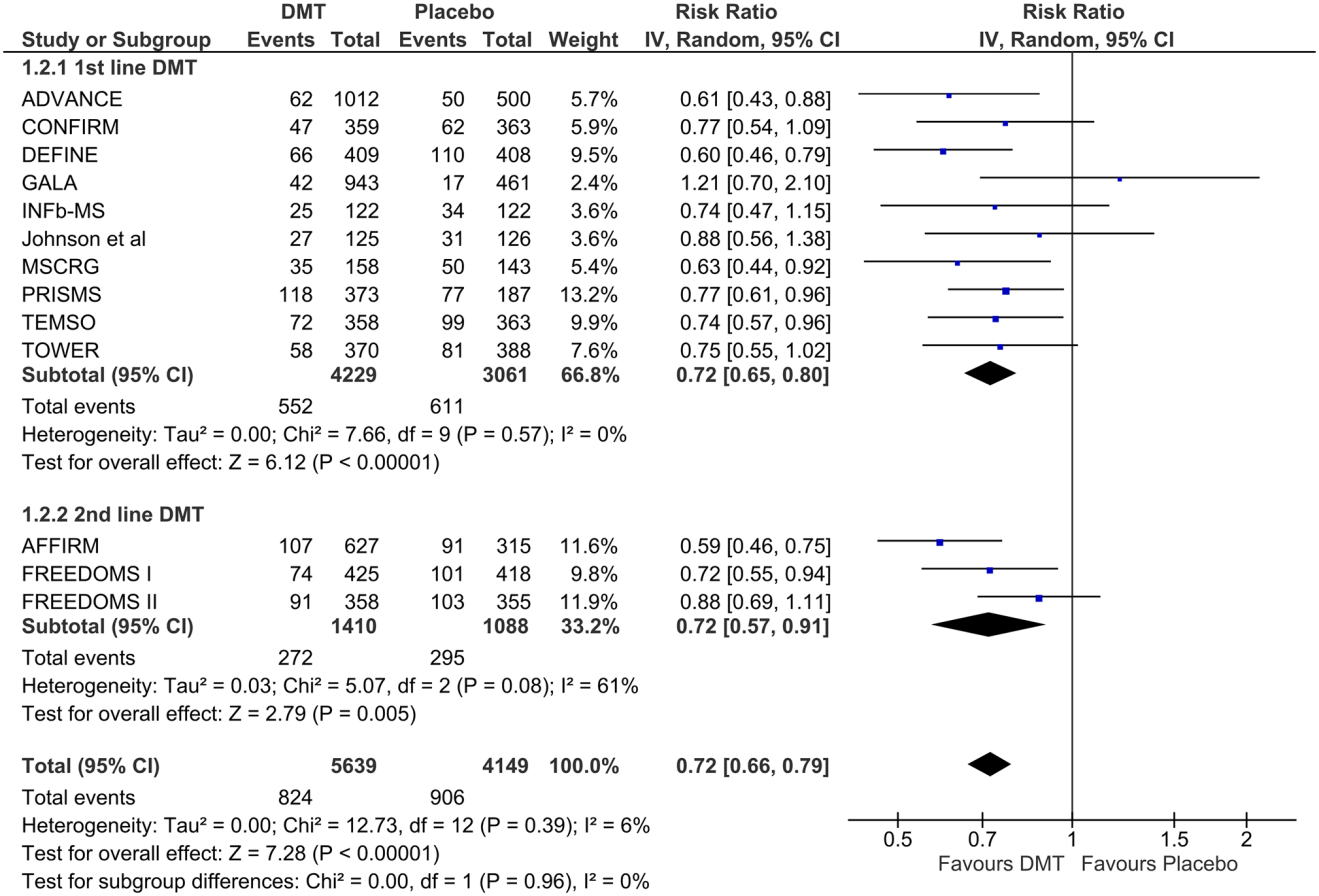
Subgroup analysis according to the current categorization of eligible disease modifying therapies as “first line” and “second line” drug options for the treatment of relapsing-remitting multiple sclerosis.

**Fig 5:**
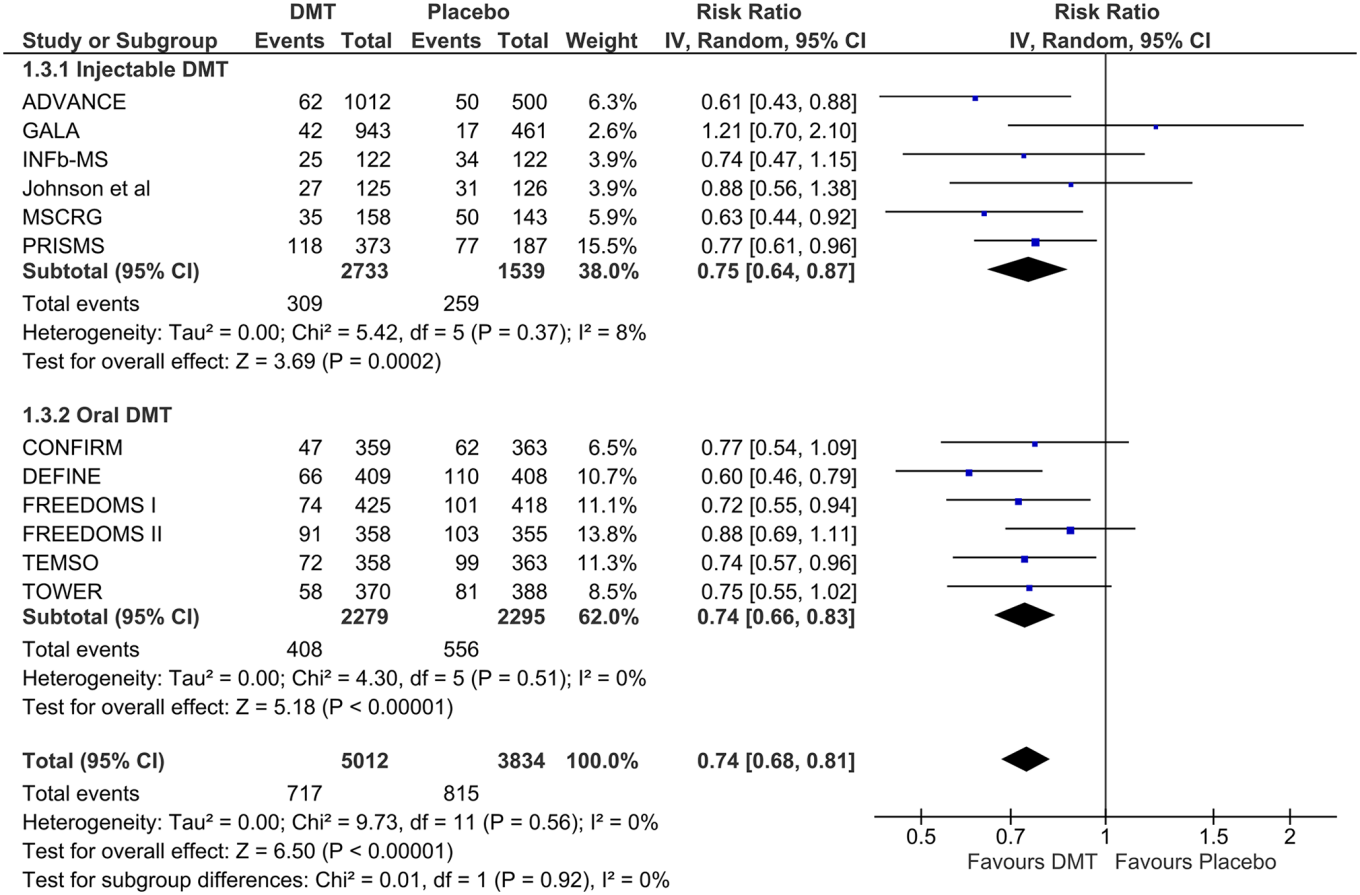
Subgroup analysis according to the route of administration (injectable vs. oral) of eligible disease modifying therapies for the treatment of relapsing-remitting multiple sclerosis.

In subsequent subgroup analyses, neither dichotomization of DMTs as “first” and “second” line RRMS therapies [RR = 0.72 (95% CI = 0.65–0.80) vs. RR = 0.72 (95% = 0.57–0.91); p = 0.96; Fig 4] nor the route of administration (injectable or oral) [RR = 0.75 (95% CI = 0.64–0.87) vs. RR = 0.74 (95% CI = 0.66–0.83); p = 0.92; Fig 5] had a differential effect on the risk of disability progression throughout each study follow-up period. In both the aforementioned analyses no evidence of substantial heterogeneity was found both within and between subgroups (p>0.05 for Cochran Q test & I^2^<75%).

In meta-regression analysis, the percentage of patients with disability progression was not found to be associated with the reported study duration (regression slope = -0.14; 95% CI: -0.39–0.11; p = 0.263; S2 Fig).

## Discussion

Our study showed that currently approved DMD for RRMS are effective in reducing disability progression compared to placebo. In all study protocols, except for two [24, 33], disability progression was assessed at 3-months and this is one of the main strengths of pooling EDSS data across trials. Moreover, we detected no significant heterogeneity in the risk reduction of disability progression across different subgroup analyses including “first” vs. “second” line DMD and oral vs. injectable route of administration.

In the pairwise comparison of a recent network meta-analysis on the currently available immunomodulator and immunosuppressive treatments for multiple sclerosis natalizumab and subcutaneous IFNß-1a were found to be significantly more effective (OR = 0.62, 95%CI:0.49–0.78 and OR = 0.35, 95%CI:0.17–0.70, respectively) than intramuscular IFNß-1a in the reduction of disability progression in patients with RRMS at 2 years follow-up. However, the confidence in this result was graded as moderate by the authors, due to the moderate quality of evidence derived from the trials [35]. Our results are not directly comparable to this network meta-analysis since our aim was not to compare individual DMD against each other. Instead, we systematically evaluated potential sources of heterogeneity in the effect of DMD on disability progression using sensitivity analyses.

Our observation regarding the lack of differential effect in disability progression between “oral” and “injectable” DMD is intriguing. This finding appears to be in line with available data from individual head-to-head comparisons in RCT: (i) TRANSFORMS (Trial Assessing Injectable Interferon versus FTY720 Oral in Relapsing—Remitting Multiple Sclerosis) comparing oral fingolimod to intramuscular IFNß-1a [36], (ii) TENERE (the Terfiflunomide and Rebif study) comparing oral teriflunomide to subcutaneous IFNß-1a [37] and (iii) CONFIRM [31] (Efficacy and Safety Study of Oral BG00012 With Active Reference in Relapsing-Remitting Multiple Sclerosis) comparing oral dimethyl fumarate to subcutaneous glatiramer acetate. Interestingly, oral DMD did not reduce disability progression in comparison to the injectable therapies in any of the three trials. Similarly, our finding regarding the lack of differential effect on disability progression between “first” and “second” line DMD is not contradicted by the available data from a single RCT (TRANSFORMS) [36]. Notably, no direct comparisons were performed in the SENTINEL (Safety and Efficacy of Natalizumab in combination with Interferon Beta-1a in patients with Relapsing Remitting Multiple Sclerosis) trial between natalizumab and intramuscular IFNß-1a since the active treatment group was allocated to combination therapy with natalizumab and IFNß-1a [38].

Certain limitations need to be acknowledged in the interpretation of our study results. First, in the current systematic review and meta-analysis we evaluated only the effect of disability worsening, without reporting data on other established markers of disease activity (freedom of relapse, lack of new/enlarging T2 lesions and gadolinium-enhancing lesions on magnetic resonance imaging) [39] or brain volume loss [40]. However, in a large multicentre study both brain atrophy and lesion volumes were also found to be significant predictors of long term disability in patients with MS [41]. Likewise, progression in disability (measured with the EDSS scale) was found be directly associated with regional grey matter atrophy in a follow-up MRI evaluation study of patients with RRMS [42]. Furthermore, we have recently reported that DMD for RRMS appear to be effective in attenuating brain atrophy using a similar meta-analytical approach, while DMD benefit on brain volume loss increased linearly with longer treatment duration [11]. Second, four potentially eligible studies were excluded from the final quantitative assessment (meta-analysis) because they provided neither percentages nor numbers of patients with disability progression during the study period [18–21]. As for the included study protocols there is also an unclear risk for selection bias in 2 of them due to non adequate report in random sequence generation and allocation concealment [22, 23]. Third, most of the study protocols reported either considerable (5–20%) [22–27] or significant (>20%) [28–32] rates of loss to follow-up during the study period. Moreover, bias related to funding source is a major concern for all included studies, as they disclose financial and/or other support from the pharmaceutical industries that produce the drug under consideration in each trial. Finally, we should underline that independent studies were compared in the present meta-analysis, and thus all inferences among different DMD should be interpreted with caution.

In conclusion available DMD appear to be effective in reducing the disability progression in patients with RRMS, independent of the route of administration and their classification as “first” or “second” line therapies. However, attrition and funding source biases need to be taken into account in the interpretation of these findings.

## Supporting Information

S1 Fig Funnel plot for the risk of publication bias. (TIF)

S2 Fig Meta-regression analysis on the association between the percentage of patients with disability progression and study duration. (TIF)

S1 PRISMA Checklist PRISMA 2009 Checklist. (DOC)

S1 Table Study designs and baseline characteristics of the eligible studies included in meta-analysis. (DOC)
