# Supplementary figures and images for: The Effect of Disease Modifying Therapies on Disease Progression in Patients with Relapsing-Remitting Multiple Sclerosis: A Systematic Review and Meta-Analysis
Source: PLoS One. 2015 Dec 7;10(12):e0144538. doi: 10.1371/journal.pone.0144538 (PMC4671570; doi:10.1371/journal.pone.0144538)

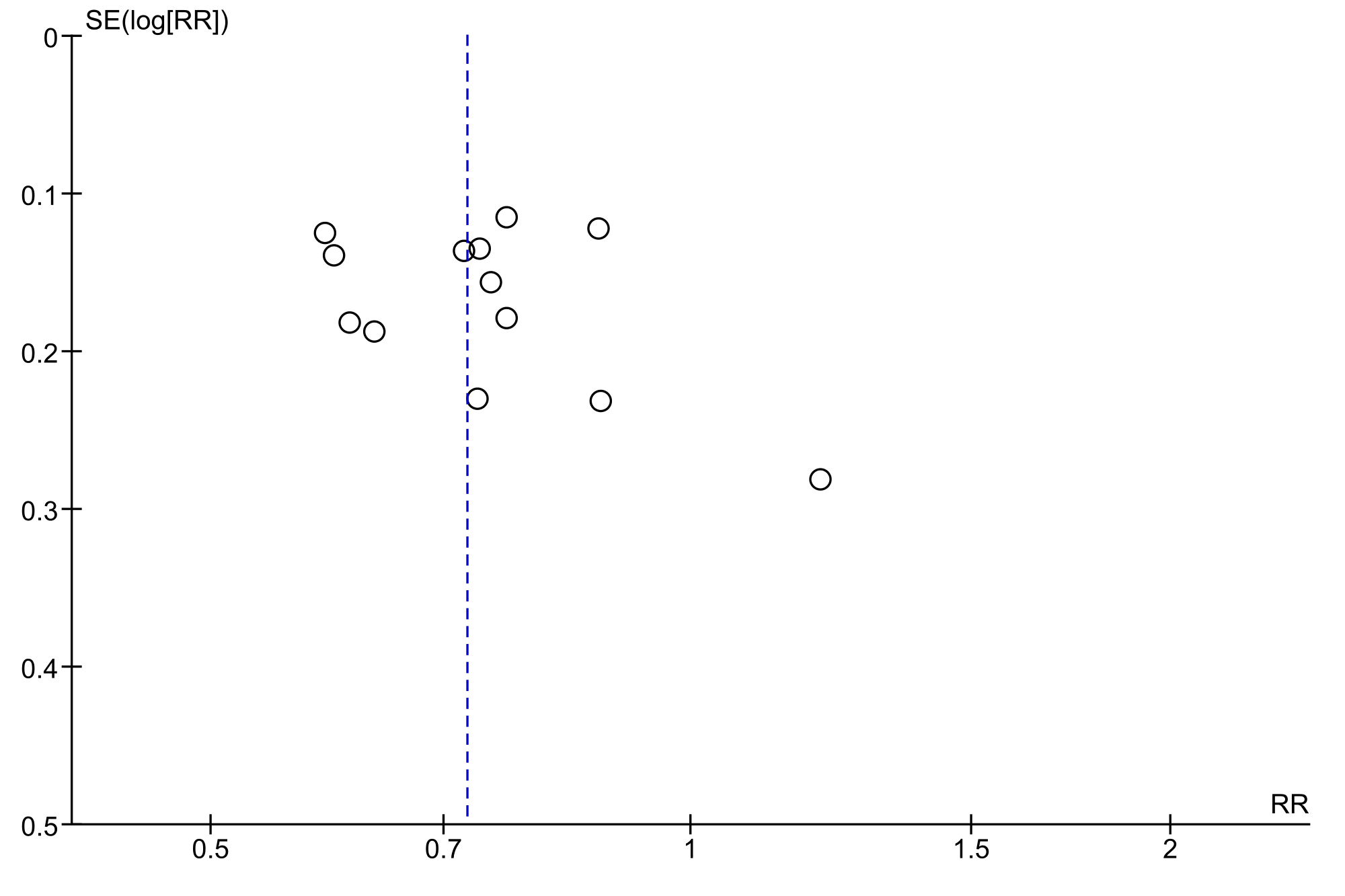

Supplement: S1 Fig — (TIF) [file pone.0144538.s001.tif]

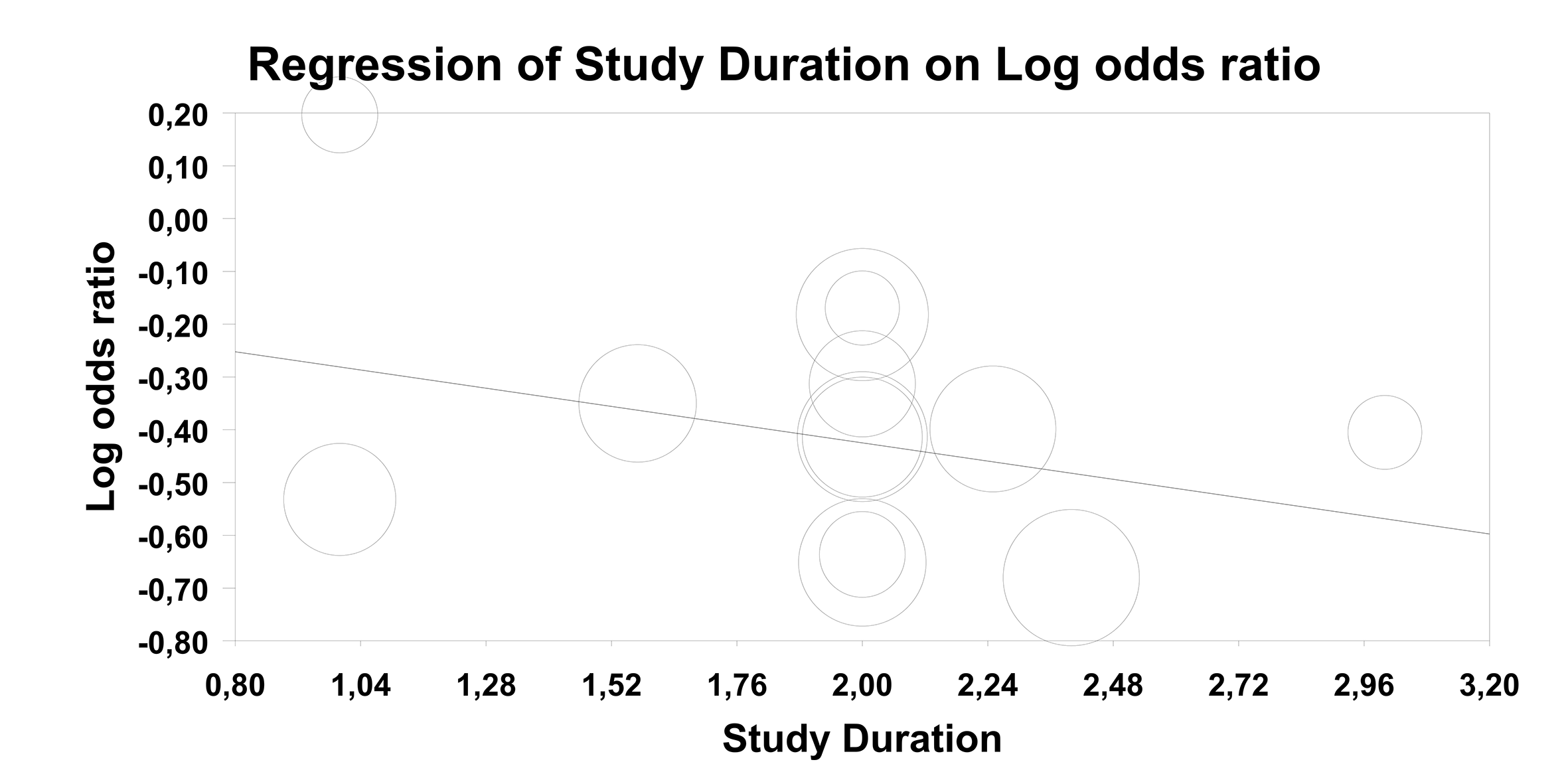

Supplement: S2 Fig — (TIF) [file pone.0144538.s002.tif]
