# Supplementary material for: The Effect of Disease Modifying Therapies on Disease Progression in Patients with Relapsing-Remitting Multiple Sclerosis: A Systematic Review and Meta-Analysis
Source: PLoS One. 2015 Dec 7;10(12):e0144538. doi: 10.1371/journal.pone.0144538 (PMC4671570; doi:10.1371/journal.pone.0144538)
Supplement: S1 Table — (DOC) [file pone.0144538.s004.doc]

**Table 1. Study designs and baseline characteristics of the eligible studies included in meta-analysis**

|  | IFNB-MS (1993)^22^ Betaferon | Copolymer (1995)^23^ Copaxone | MSCRG (1996)^24^ Avonex | PRISMS (1998)^25^ Rebif | AFFIRM  (2006)^26^  Tysabri | FREEDOMS (2010)^27^ Gilenya | FREEDOMS II (2014)^28^ Gilenya | TEMSO (2011)^29^ Aubagio | TOWER (2014)^30^ Aubagio | CONFIRM (2012)^31^ Tecfidera | DEFINE  (2012)^32^  Tecfidera | **GALA**  **(2013)^33^**  **Copaxone** | **ADVANCE (2014)^34^ Plegridy** |
| --- | --- | --- | --- | --- | --- | --- | --- | --- | --- | --- | --- | --- | --- |
| No of patients underwent randomization | 372 | 251 | 301 | 560 | 942 | 1272 | 1083 | 1088 | 1169 | 1430 | 1237 | **1404** | **1512** |
| **Study design** |  |  |  |  |  |  |  |  |  |  |  |  |  |
| Diagnostic criteria | Poser | Poser | Poser | Poser | McDonald 2001 | McDonald 2005 | McDonald 2005 | McDonald 2001 | McDonald 2005 | McDonald 2005 | McDonald 2005 | **McDonald 2005** | **McDonald 2005** |
| Age (yr) | 18-50 | 18-45 | 18-55 | Adults | 18-50 | 18-55 | 18-55 | 18-55 | 18-55 | 18-55 | 18-55 | **18-55** | **18-65** |
| Disease activity | ≥ 2 relapses in the previous 2 yr | ≥ 2 relapses in the previous 2 yr | ≥ 2 relapses in the previous 3 yr | ≥ 2 relapses in the previous 2 yr | ≥1 relapse in the previous 12mo | ≥ 1 relapse in the previous 1yr or ≥ 2 relapses in the previous 2yr | ≥ 1 relapse in the previous 1yr or ≥ 2 relapses in the previous 2yr | ≥ 2 relapses in the previous 2yr or ≥ 1 relapse in the previous 1yr | ≥ 2 relapses in the previous 2yr or ≥ 1 relapse in the previous 1yr | ≥1 relapse in within 12 mo, or ≥ 1 Gd(+) within 6 wk | ≥1 relapse within 12 mo, or ≥ 1 Gd(+) within 6 wk | **≥ 1 relapse in the previous 1yr or ≥ 2 relapses in the previous 2yr, or 1 relapse between 1 and 2 years + 1 Gd(+) within 1 year** | **≥ 2 relapses in the previous 3yrs, with 1 within 1yr** |
| MS subtype | RRMS | RRMS | RRMS | RRMS | RRMS | RRMS | RRMS | RRMS (91.5%)  SPMS (4.7%)  PRMS (3.8%) | RRMS (97.5%)  SPMS (0.8%)  PRMS (1.7%) | RRMS | RRMS | **RRMS** | **RRMS** |
| EDSS inclusion criteria | 0-5.5 | 0-5 | 1-3.5 | 0-5 | 0-5 | 0-5.5 | 0-5.5 | 0-5.5 | 0-5.5 | 0-5 | 0-5 | **0-5.5** | **0-5** |
| Primary end point | ARR, Proportion relapse-free patients | Relapse Rate (24 mo), ARR,  Relapses-No (24 mo) | Time to sustained disability progression | Relapse count (2yr) | EDSS progression (2y), ARR | ARR | ARR | ARR | ARR | ARR | Proportion of patients with relapse at 2 yr | **Relapse count (1y)** | **ARR** |
| EDSS progression definition | 1.0 point (3mo) | 1.0 point (3mo) | 1.0 point (6mo) | 1.0 point (3mo) | 1.0 point (for EDSS≥1), 1.5 point (for EDSS=0) (12 weeks) | 1.0 point (0.5 point EDSS=5.5)  (3mo) | 1.0 point (0.5 point EDSS>5.0)  (3mo) | 1.0 point (or ≥0.5 for EDSS>5.5) (12 weeks) | 1.0 point (or ≥0.5 for EDSS>5.5) (12 weeks) | 1.0 point (for EDSS ≥1.0), 1.5 points (for EDSS=0) (12 weeks) | 1.0 point (for EDSS ≥1.0), 1.5 points (for EDSS=0) (12 weeks) | **1.0 point**  **(12 mo)** | **1.0 point (for EDSS ≥1.0), 1.5 points (for EDSS=0) (12 weeks)** |
| Treatment history | 0% | 0% | 0% | 0% | Unclear | 59.1% | 74.8% | 27.0% | 32.8% | 29% | 40.7% | **13.6%** | **18.8%** |
| Study duration | 3yr | 24 mo | 2 yr | 2 yr | 116 weeks | 24 mo | 24 mo | 108 weeks | 575 days (median) | 96 weeks | 96 weeks | **1yr** | **1yr** |
| **Demographic characteristics** |  |  |  |  |  |  |  |  |  |  |  |  |  |
| Age, Mean ± SD (yr) | 35.5±7 | 34.4±6.2 | 36.8±7.4 | 34.9 (median) | 36±8.3 | 37.1±8.8 | 40.5±8.6 | 37.8±8.7 | 37.9±9.3 | 37.3±9.3 | 38.5±9.0 | **37.6±9.4** | **36.5±9.8** |
| Female (%) | 69.6% | 73.3% | 73.4% | 69% | 70% | 69.9% | 77.9% | 72.2% | 71.1% | 69.4% | 73.6% | **67.9%** | **71%** |
| White race (%) | 93.5% | 94% | 92.4% | NR | 95% | NR | NR | 97.2% | 82.1% | 83.3% | 78.5% | **97.7%** | **82%** |
| Time from first MS symptom, Mean ± SD (yr) | NR | 6.9±5.0 | 6.5±5.8 | 5.3 (median) | 5 (median) | 8.2±6.6 | 10.6±8 | 8.7±6.9 | 8.0±6.7 | NR | NR | **7.7±6.6** | **6.6±6.7** |
| EDSS, Mean ± SD | 2.9±1.1 | 2.6±1.3 | 2.3±0.8 | 2.5±1.2 | 2.3±1.2 | 2.4±1.3 | 2.4±1.3 | 2.7±1.3 | 2.7±1.4 | 2.6±1.2 | 2.4±1.2 | **2.8±1.2** | **2.5±1.2** |
| Relapses-no within previous yr, Mean ± SD | 2yr: 3.4±1.6 | 2y: 2.9±1.2 | 1.2±0.6 | 2y: 3.0±1.2 | 1.52±0.86 | 1.5±0.8 | 1.5±0.9 | 1.4±0.7 | 1.4±0.7 | 1.4±0.7 | 1.3±0.7 | **1.3±0.6** | **1.6±1.3** |
| No of Gd(+),Mean ± SD | NR | NR | 2.76±6.05 | NR | 2.2±4.7 | 1.6±4.4 | 1.3±3.4 | 1.7±4.2 | NR | NR | 1.3±3.6 | **1.6±5.6** | **1.5±6.9** |

Abbreviations: No: number, yr: year(s), NR: not reported, SD: standard deviation, ARR: annualized relapse rate, Gd(+): Gadolinium enhancing lesions, RRMS: Relapsing-Remitting MS, SPMS: Secondary progressive MS, PRMS: Progressive relapsing MS
